# Supplementary material for: Diabatic heating governs the seasonality of the Atlantic Niño
Source: Nat Commun. 2021 Jan 14;12:376. doi: 10.1038/s41467-020-20452-1 (PMC7809448; doi:10.1038/s41467-020-20452-1)
Supplement: Supplementary file 1 — Supplemetary Information File [file 41467_2020_20452_MOESM1_ESM.pdf]

Supplementary Information for

Diabatic heating governs the seasonality of the Atlantic Niño

Hyacinth C. Nnamchi[1,2,*], Mojib Latif[1], Noel S. Keenlyside[3], Joakim Kjellsson[1] and Ingo Richter[4]

[1]GEOMAR Helmholtz Centre for Ocean Research Kiel, Kiel, Germany

[2]Department of Geography, University of Nigeria, Nsukka, Nigeria

[3]Geophysical Institute, University of Bergen, and Bjerknes Centre for Climate Research, Bergen, Norway

[4]Application Laboratory, JAMSTEC, Yokohama, Japan

*Hyacinth Nnamchi (hnnamchi@geomar.de; hyacinth.nnamchi@unn.edu.ng)

Supplementary Table 1. Satellite and *in situ* derived observations

Data	Resolution	Reference	Download Link
EN4 v4.2.1	1°×1°×42L	Good et al.[1]	https://www.metoffice.gov.uk/hadobs/en4
OAFLUX	1°×1	Yu & Weller[2]	http://apdrc.soest.hawaii.edu/dods/public_data/WHOI_OAFlux
OLR	2.5°×2.5°	Liebmann & Smith[3]	https://psl.noaa.gov/data/gridded/data.interp_OLR.html
Precipitation	2.5°×2.5°	Adler et al.[4]	https://www.esrl.noaa.gov/psd/data/gridded/data.gpcp.html
SSH	0.25°×0.25°	Rio et al. [5]	https://marine.copernicus.eu/
SST	0.25°×0.25°	Reynolds et al.[6]	https://www.esrl.noaa.gov/psd/data/gridded/data.gpcp.html

Supplementary Table 2. Atmospheric reanalysis data sets

	Data	Resolution	Reference	Download Link
1.	CFSR	1°×1°×37L	Saha et al.[7]	https://rda.ucar.edu/datasets/ds093.1/
2.	ERA5	0.25°×0.25° ×137L	Hersbach et al.[8]	https://www.ecmwf.int/en/forecasts/datasets/reanalysis-datasets/era5
3.	ERAI	0.75°×0.75° ×60L	Dee et al.[9]	https://www.ecmwf.int/en/forecasts/datasets/ reanalysis-datasets/era-interim
4.	JRA55	1.25°×1.25° ×37L	Harada et al.[10]	https://jra.kishou.go.jp/JRA-55/index_en.html
5.	MERRA2	0.50°×0.62° ×42L	Molod et al.[11]	https://gmao.gsfc.nasa.gov/reanalysis/MERRA-2/

Supplementary Table 3. Ocean reanalysis data sets

	Data	Resolution	Ocean model	Atmospheric forcing	Reference	Download Link
1.	GECCO2	1°×0.3°×50 L	MITgcm	NCEP1	Köhl[12]	https://icdc.cen.uni-hamburg.de/1/daten/reanalysis-ocean/gecco2.html
2.	GODAS	0.4°×1°×42 L	MOM v3	NCEP2	Behringer et al.[13]	https://www.esrl.noaa.gov/psd/data/gridded/data.godas.html
3.	ORAS4	1°×1°×42L	NEMO v3.0	ERA40, ERA-Interim	Balmaseda et al.[14]	http://icdc.cen.uni-hamburg.de/projekte/easy-init/easy-init-ocean.html
4.	ORAS5	0.25°×0.25 °×75L	NEMO v3.4	ERA-Interim	Zuo et al.[15]	http://icdc.cen.uni-hamburg.de/projekte/easy-init/easy-init-ocean.html
5.	SODA3 v3.4.2	0.25°×0.25 °×50L	MOM v5.1	ERA-Interim	Carton et al.[16]	https://www2.atmos.umd.edu/~ocean/index_files/soda3.4.2_mn_download_b.htm

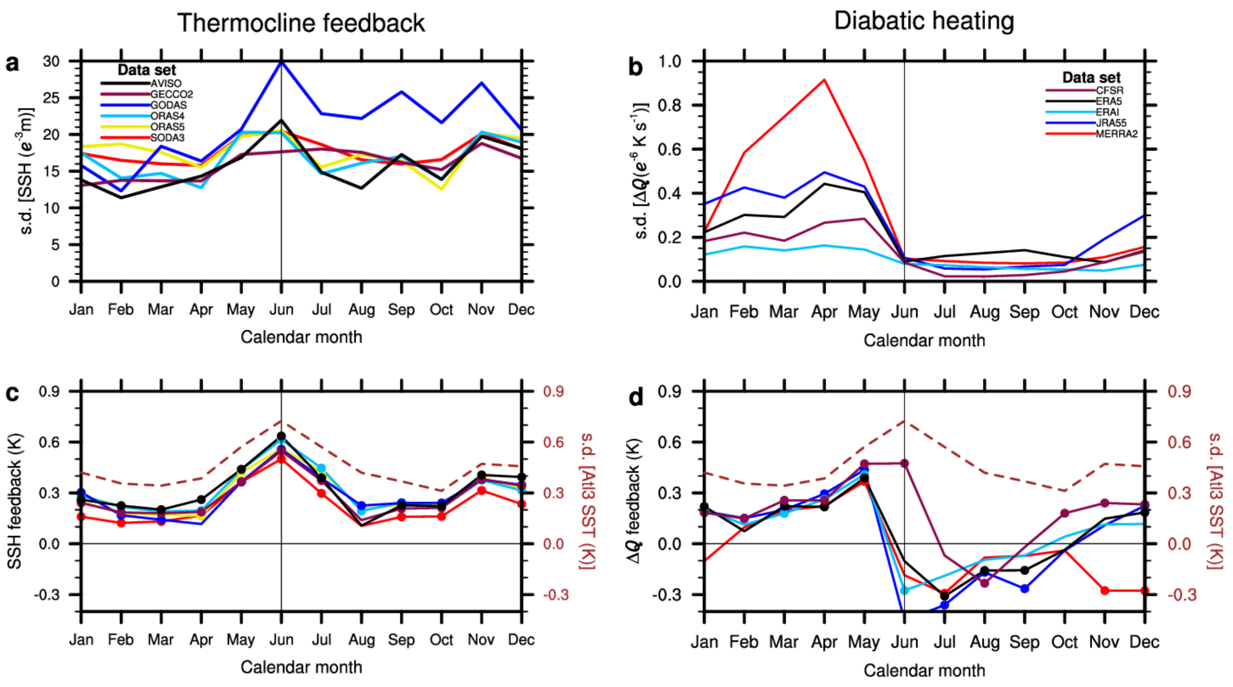

Supplementary Fig. 1: Same as Figs. 1a-d but for the Atlantic Niño region. Standard deviations of seasonally stratified **a** sea surface height (SSH) and **b** diabatic heating gradient (ΔQ) averaged over the Atl3 region (3°N–3°S, 0°–20°W) in multiple data sets. **c** (left scale) Local thermocline feedback (K) calculated as the Atl3 SST (that is, average in the region 3°N–3°S, 0°–20°W) regressed on the normalized SSH averaged in that region for each calendar month. **d** (left scale) Local diabatic heating feedback calculated as the Atl3 SST regressed on the normalized ΔQ averaged in that region for each calendar month. In both **c** and **d,** the right scale shows the seasonally stratified standard deviations of the Atl3 SST (K), circular ticks denote statistical significance at the 95% confidence level.

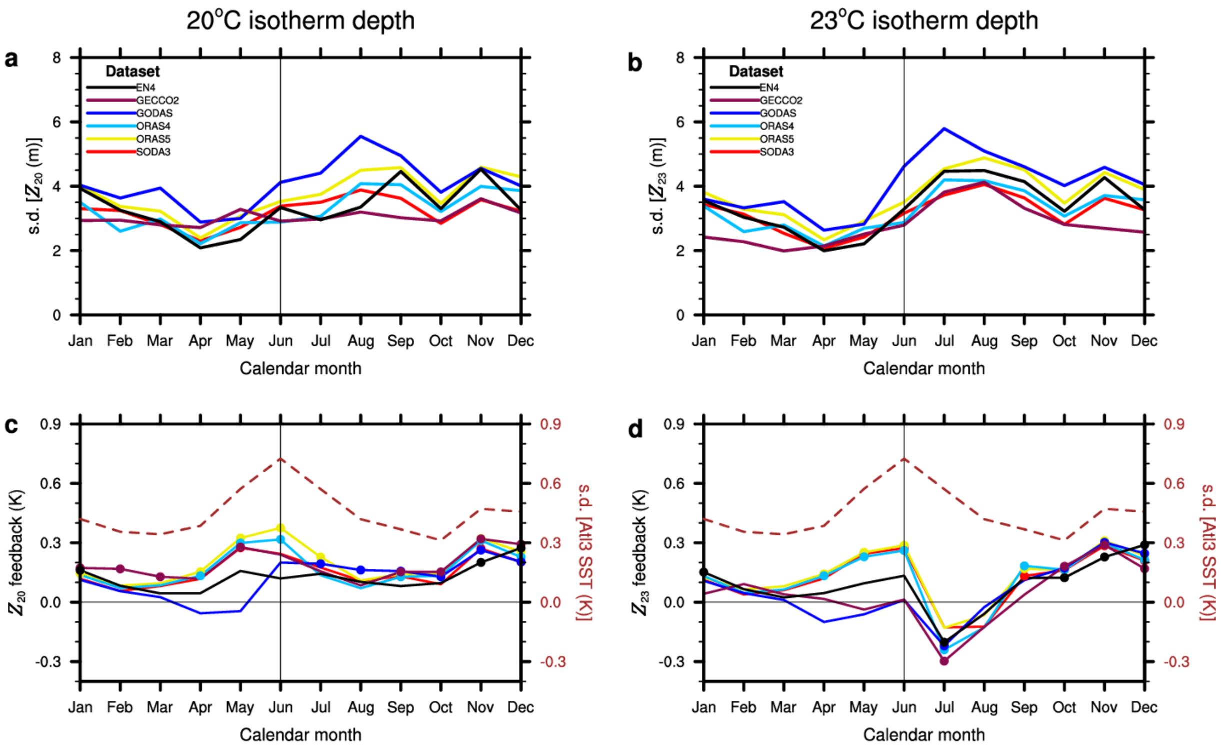

Supplementary Fig. 2: Thermocline feedback based on the isotherm definitions. Standard deviations of seasonally stratified **a** 20°C isotherm depth (Z_{20}) and **b** 23°C isotherm depth (Z_{23}) averaged over the equatorial Atlantic region (3°N–3°S, 5°E–40°W) in multiple data sets. Thermocline feedback is here defined as the Atl3 SST index (that is, average in the region 3°N–3°S, 0°–20°W) regressed on the normalized basin indices of **c** Z_{20} and **d** Z_{23} in different calendar months. Circular ticks in **c** and **d** denote statistical significance at the 95% confidence level.

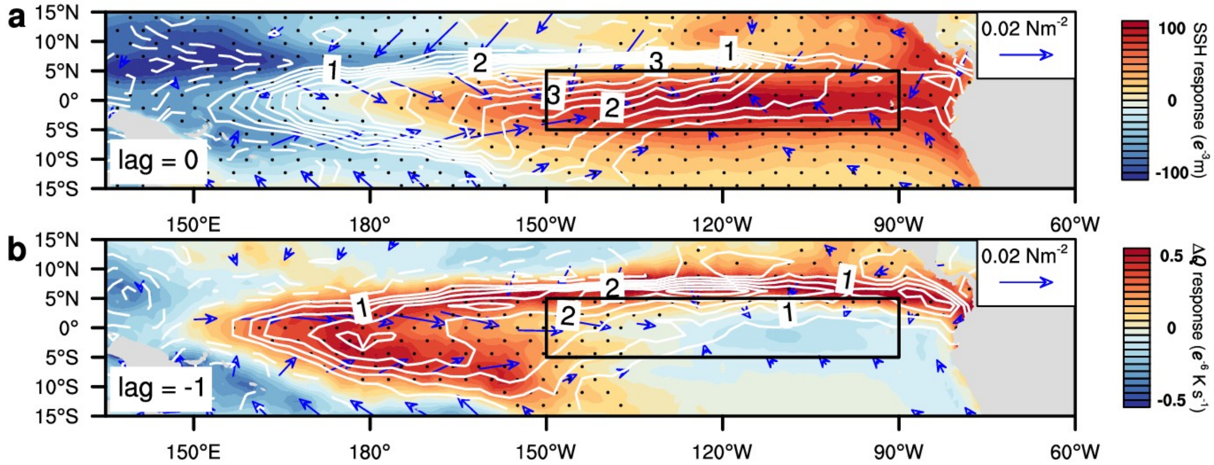

Supplementary Fig. 3: Pacific El Niño-type thermocline and diabatic heating. The colour scales show the anomalies of **a** sea surface height (SSH), **b** diabatic heating gradient (ΔQ), wind stress (only statistically significant vectors are plotted) and precipitation (white contours, at interval of 0.5 mm day^{-1}) in **a** December and **b** November regressed on the normalized Niño3 index (that is, SST average in the region 5°N–5°S, 90°–150°W) during the peak month of December. The SSH, ΔQ and wind stress are based on the ensemble-means; stippling denotes statistical significance at the 95% confidence level.

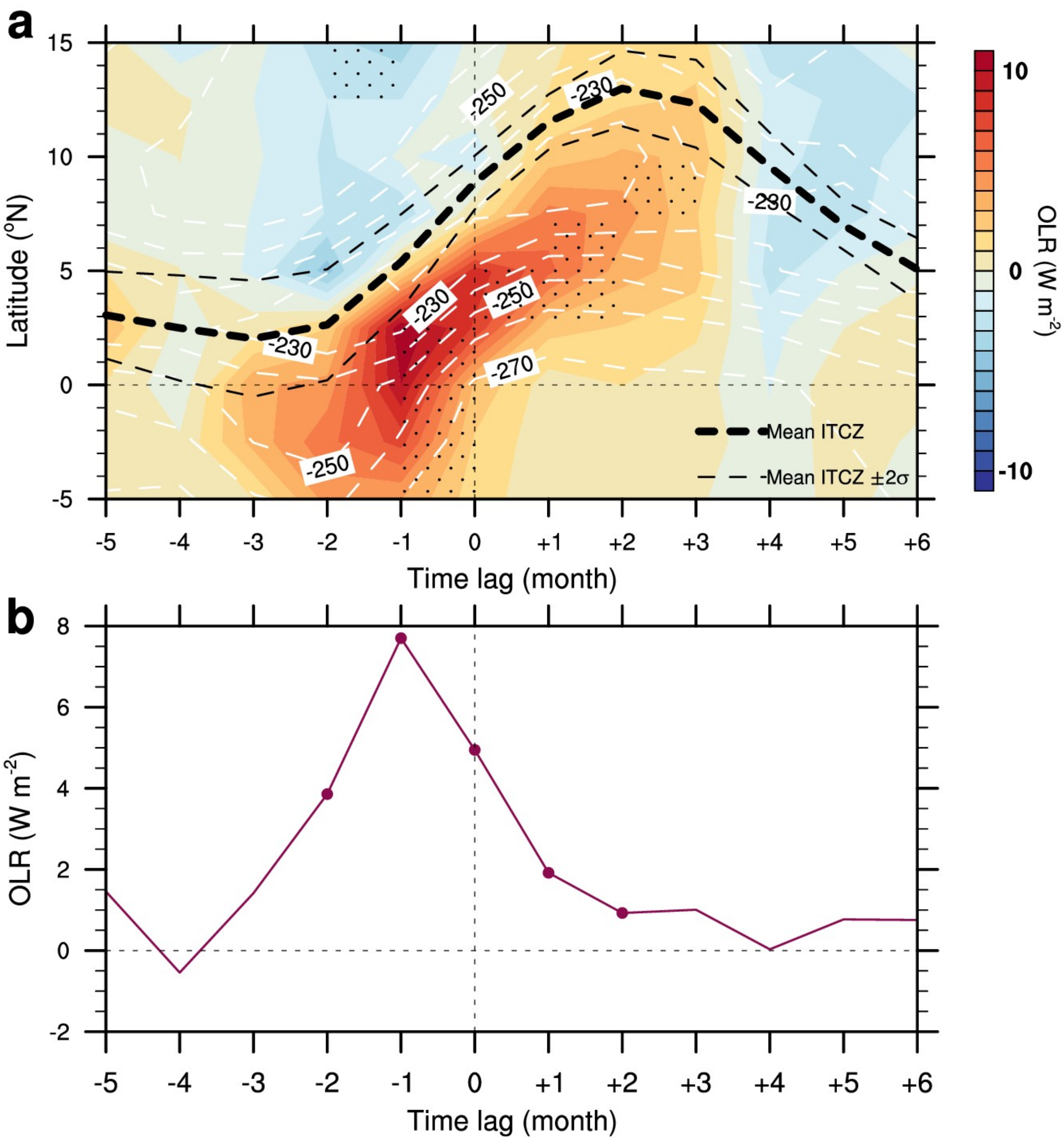

Supplementary Fig. 4: Related outgoing longwave radiation variability. a Seasonally stratified outgoing longwave radiation (OLR) anomalies along 20°W representing the western edge of the Atlantic Niño region regressed on the June (lag=0) normalized Atl3 SST index (that is, average in the region 3°N–3°S, 0°–20°W). Stipples denote statistical significance at the 95% confidence level. The white contours show the climatological-mean OLR. The dashed black curves denote the mean inter-tropical convergence zone (ITCZ) defined by the latitude of zero meridional wind stress (thick curve) and its two-standard deviations represented by ±2σ (thin curves). **b** OLR anomalies averaged over the equatorial Atlantic region (3°N–3°S, 5°E–40°W) in all months regressed on the normalized Atl3 SST index fixed in June (lag=0). Circular ticks in **b** denote statistical significance at the 95% confidence level.

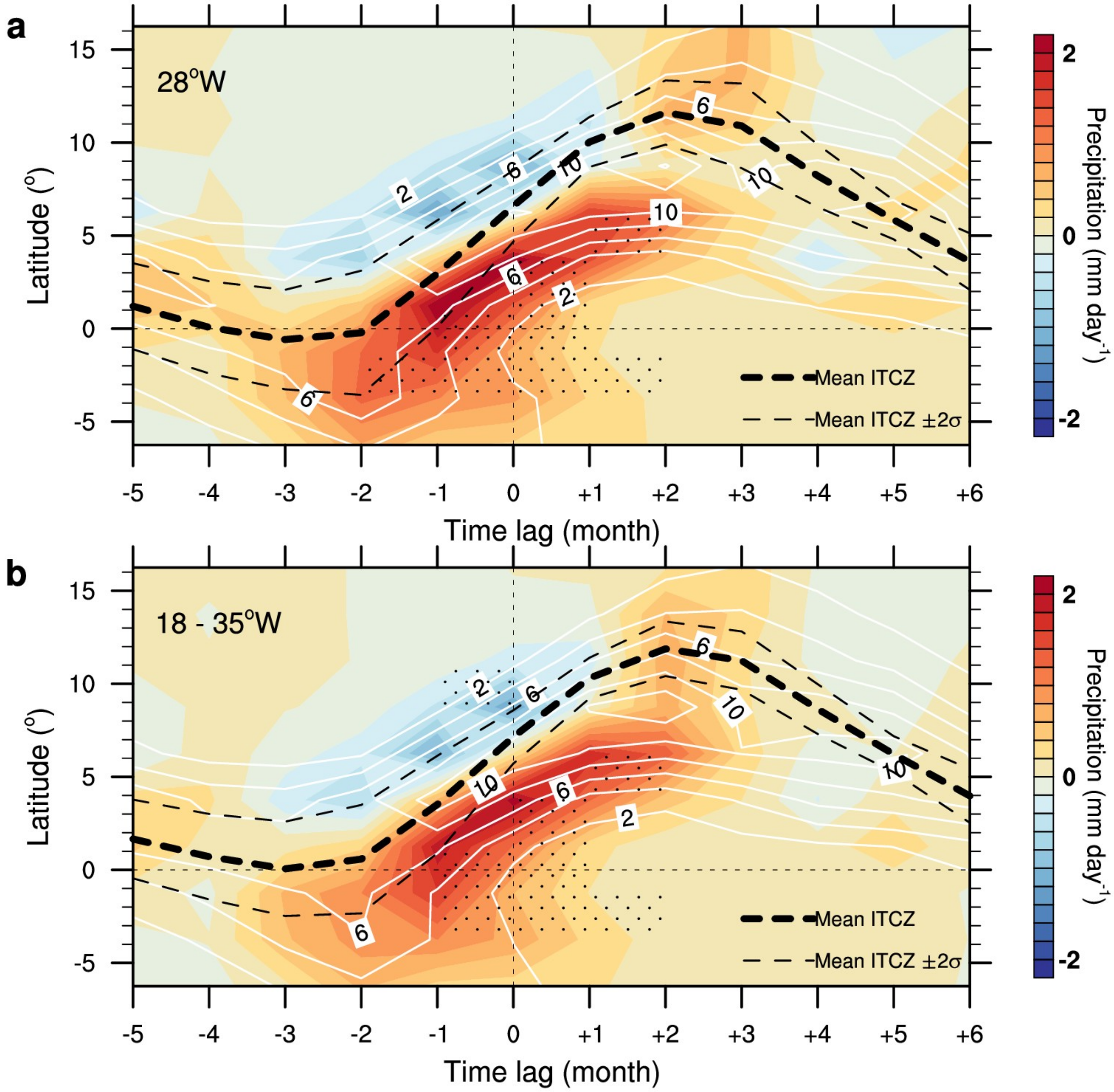

Supplementary Fig. 5: Related precipitation at 28°W and 18-35°W. Seasonally stratified precipitation anomalies along **a** 28°W and **b** 18-35°W regressed on the June (lag=0) normalized Atl3 SST index (that is, average in the region 3°N–3°S, 0°–20°W). The stipples denote statistical significance at the 95% confidence level. The solid white contours show the climatological-mean precipitation. The dashed black curves denote the mean inter-tropical convergence zone (ITCZ) defined by the latitude of zero meridional wind stress (thick curve) and its two-standard deviations represented by ±2σ (thin curves).

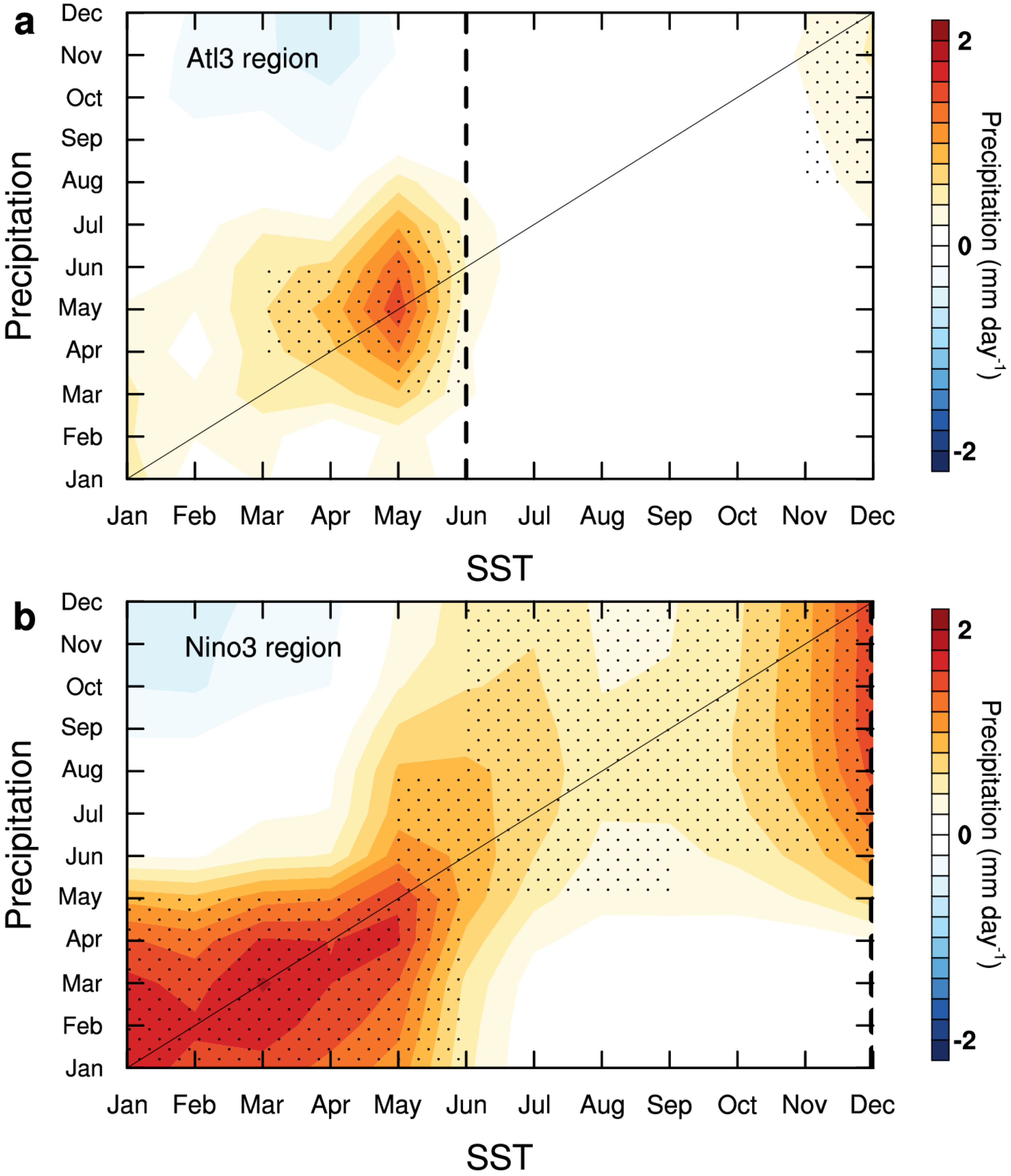

Supplementary Fig. 6: Precipitation as a function of El Niño indices. Precipitation anomalies averaged over the **a** Atl3 (3°N–3°S, 0°–20°W) and **b** Niño3 (5°N–5°S, 90°–150°W) regions in all calendar months regressed on the normalized sea surface temperature (SST) indices in all calendar months in the respective regions. The thin solid line denotes the regression for the same month. In each panel, the dashed vertical line represents the month in **a** June and **b** December of maximum SST variability.

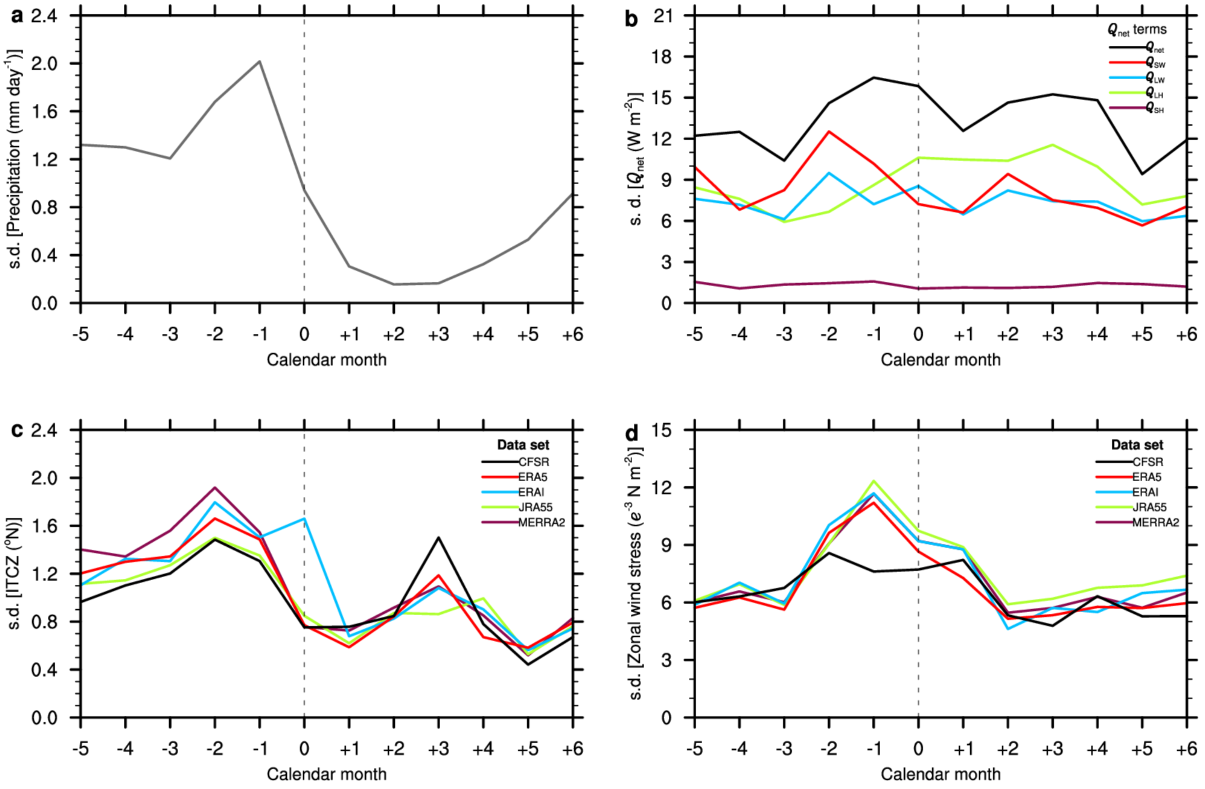

Supplementary Fig. 7: Atmospheric variability in the equatorial Atlantic. Seasonally stratified standard deviations of the indices of **a** precipitation averaged over the equatorial Atlantic (3°S–3°N, 5°E–40°W) and **b** surface net heat flux (Q_{net}) and its components (surface net shortwave Q_{sw}, surface net longwave Q_{sw}, latent Q_{lh} and sensible Q_{sh} heat fluxes) averaged over the Atl3 region (3°S–3°N, 0°–20°W). **c,d** Standard deviations of the **c** inter-tropical convergence zone (ITCZ) defined as the latitude of zero meridional wind stress along longitude 20°W over tropical Atlantic (5°S–20°N) and **d** the zonal wind stress averaged over the western equatorial Atlantic (3°S–3°N, 20°–40°W).

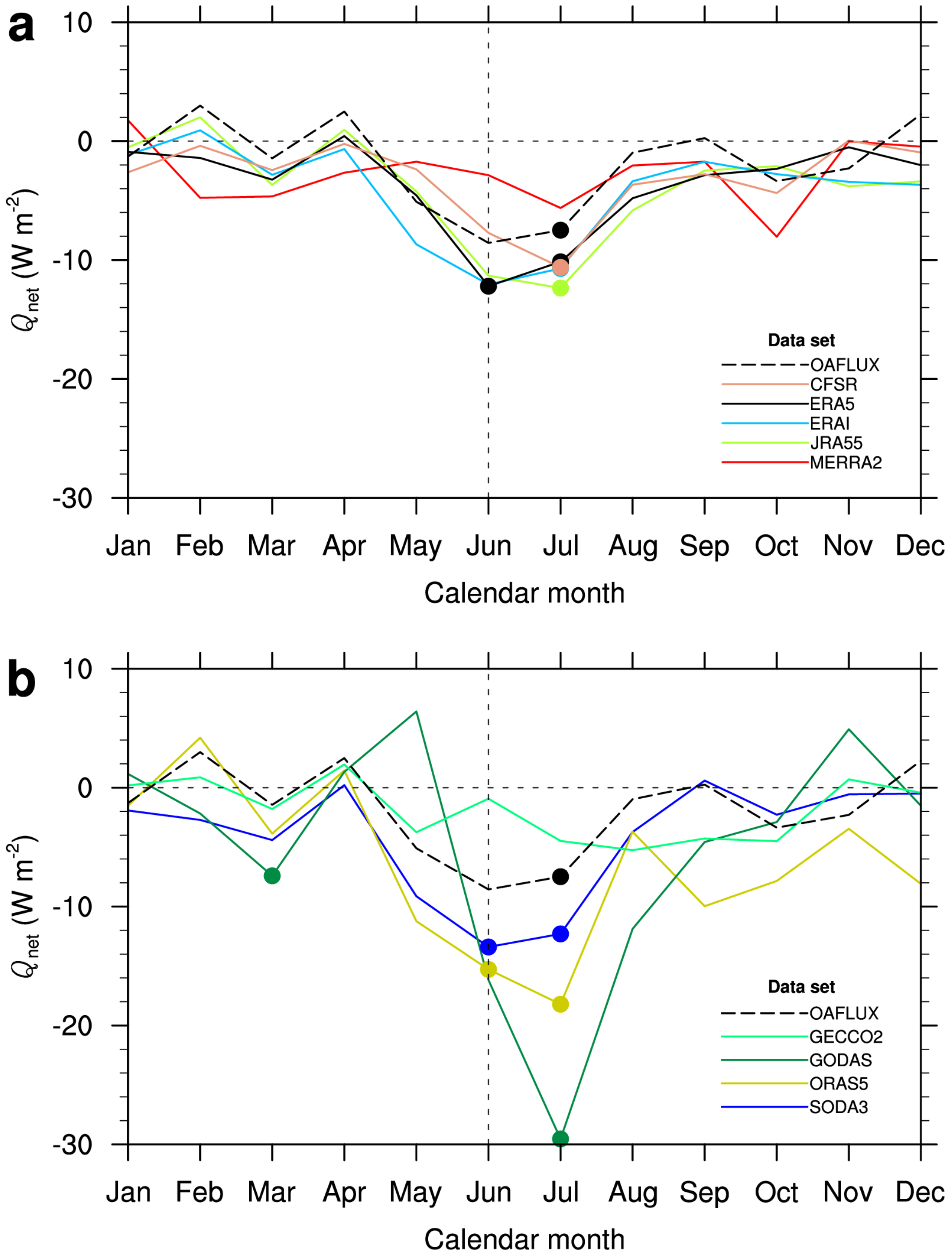

Supplementary Fig. 8: Composite of the surface net heat flux. The curves shows the surface net heat flux (Q_{net}) from **a** atmospheric and **b** oceanic reanalysis systems are averaged over the Atl3 region (3°S–3°N, 0°–20°W). The composites are for the Atlantic Niño years discussed in Methods. Circular ticks denote statistical significance at the 95% confidence level. Note that Q_{net} is not available in the ORAS4 archive.

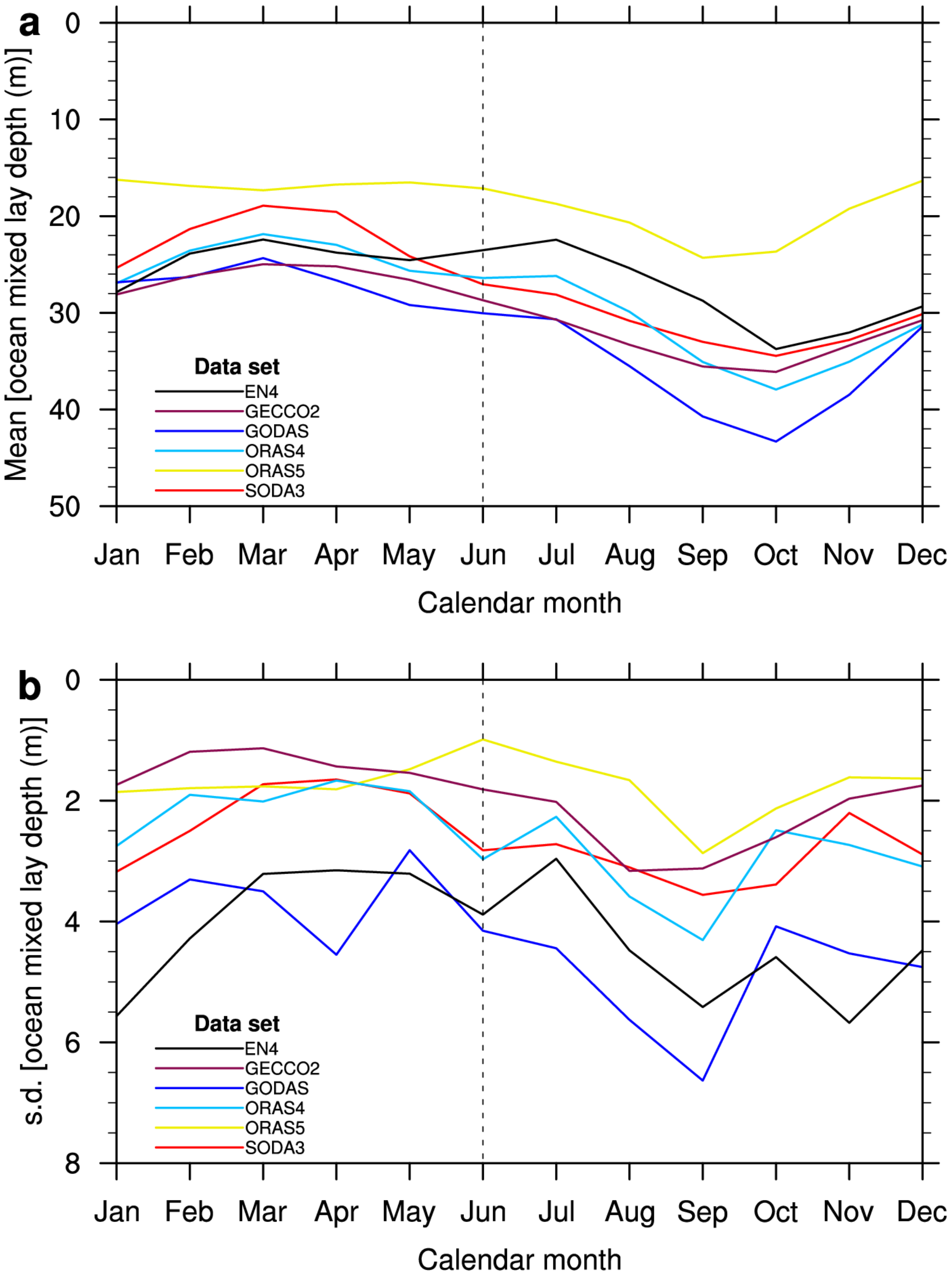

Supplementary Fig. 9: Ocean mixed layer depth in the Atlantic Niño region. a Mean and **b** standard deviations of the mixed layer depth averaged over the Atl3 region (3°S–3°N, 0°–20°W).

Supplementary Referees

1. Good, S. A., Martin, M. J. & Rayner, N. A. EN4: Quality controlled ocean temperature and salinity profiles and monthly objective analyses with uncertainty estimates. *J. Geophys. Res. Ocean.* **118,** 6704–6716 (2013).
2. Yu, L. & Weller, R. A. Objectively Analyzed Air–Sea Heat Fluxes for the Global Ice-Free Oceans (1981–2005). *Bull. Am. Meteorol. Soc.* **88,** 527–540 (2007).
3. Liebmann, B. & Smith, C. A. Description of a Complete (Interpolated) Outgoing Longwave Radiation Dataset. *Bull. Am. Meteorol. Soc.* **77,** 1275–1277 (1996).
4. Adler, R. F. et al. The Version-2 Global Precipitation Climatology Project (GPCP) Monthly Precipitation Analysis (1979–Present). *J. Hydrometeorol.* **4,** 1147–1167 (2003).
5. Rio, M.-H., Mulet, S. & Picot, N. Beyond GOCE for the ocean circulation estimate: Synergetic use of altimetry, gravimetry, and in situ data provides new insight into geostrophic and Ekman currents. *Geophys. Res. Lett.* **41,** 8918–8925 (2014).
6. Reynolds, R. W., Rayner, N. A., Smith, T. M., Stokes, D. C. & Wang, W. An Improved In Situ and Satellite SST Analysis for Climate. *J. Clim.* **15,** 1609–1625 (2002).
7. Saha, S. et al. The NCEP Climate Forecast System Version 2. J. Clim. 27, 2185–2208 (2014).
8. Hersbach, H. et al. The ERA5 global reanalysis. *Q. J. R. Meteorol. Soc.* **146,** 1999– 2049 (2020).
9. Dee, D. P. et al. The ERA-interim reanalysis: configuration and performance of the data assimilation system. *Q. J. R. Meteorol. Soc.* **137,** 553–597 (2011).
10. Kobayashi, S. et al. The JRA-55 reanalysis: General specifications and basic characteristics. *J. Meteorol. Soc. Japan.* **Ser. II 93,** 5–48 (2015).
11. Molod, A., Takacs, L., Suarez, M. & Bacmeister, J. Development of the GEOS-5 atmospheric general circulation model: evolution from MERRA to MERRA2. *Geosci. Model Dev.* **8,** 1339–1356 (2015).
12. Köhl, A. Evaluation of the GECCO2 ocean synthesis: transports of volume, heat and freshwater in the Atlantic. *Q. J. R. Meteorol. Soc.* **141,** 166–181 (2015).
13. Behringer, D. W., Ji, M. & Leetmaa, A. An Improved Coupled Model for ENSO Prediction and Implications for Ocean Initialization. Part I: The Ocean Data Assimilation System. *Mon. Weather Rev.* **126,** 1013–1021 (1998).
14. Balmaseda, M. A., Mogensen, K. & Weaver, A. T. Evaluation of the ECMWF ocean reanalysis system ORAS4. *Q. J. R. Meteorol. Soc.* **139,** 1132–1161 (2013).

15. Zuo, H., Balmaseda, M. A., Tietsche, S., Mogensen, K. & Mayer, M. The ECMWF operational ensemble reanalysis--analysis system for ocean and sea ice: a description of the system and assessment. *Ocean Sci.* **15,** 779–808 (2019).

16. Carton, J. A., Chepurin, G. A. & Chen, L. SODA3: A New Ocean Climate Reanalysis. *J. Clim.* **31,** 6967–6983 (2018).
